# Experimental Determination of Isothermal Sections in the Ni–Al–Cr–Ru Quaternary System: Implications for Ni-Based Superalloys and High-Entropy Alloys

**DOI:** 10.3390/ma19081669

**Published:** 2026-04-21

**Authors:** Jianping Huang, Dupei Ma, Zhi Li, Yan Liu, Ruihua Wang, Huayu Xiao, Qiang Zhang

**Affiliations:** 1 School of Materials Science and Engineering, Hunan Institute of Technology, Hengyang 421002, China; hjphit_1983@163.com (J.H.); 15002372590@163.com (H.X.); 2 School of Materials Science and Engineering, Xiangtan University, Xiangtan 411105, China; lizhiclsj@xtu.edu.cn (Z.L.); xiaoniudun@xtu.edu.cn (Y.L.); 17734607985@163.com (R.W.); 3 School of Materials Science and Engineering, Harbin Institute of Technology, Harbin 150001, China; 4 State Key Laboratory of Precision Welding & Joining of Materials and Structures, School of Materials Science and Engineering, Harbin Institute of Technology, Harbin 150001, China

**Keywords:** high-entropy alloy, equilibrium alloy method, CALPHAD, phase diagram, Ni-based superalloy, Ni–Al–Cr–Ru system

## Abstract

**Highlights:**

- Identification of a four-phase equilibrium region, i.e., Bcc(Cr) + β-(Ni,Ru)Al + Al_8_Cr_5_ + Al_2_Ru at 1423 K (55 at.% Al).
- Discovery that Cr addition promotes complete mutual solubility between NiAl and AlRu phases at 1173 K.
- Mapping of a wide (Ni,Ru)Al + Fcc(Ni) region at 1423 K (60 at.% Ni), valuable for alloy design.
- Offers experimental benchmarks for thermodynamic modeling (CALPHAD) of the Ni–Al–Cr–Ru system.

**Abstract:**

The phase equilibria of the Ni–Al–Cr–Ru quaternary system were systematically investigated using the equilibrated alloy method combined with scanning electron microscopy (SEM), energy-dispersive spectroscopy (EDS), and X-ray diffraction (XRD). This study focuses on three key isothermal sections within the system: 55 at.% Al at 1423 K, 55 at.% Ni at 1173 K, and 60 at.% Ni at 1423 K. In the 55 at.% Al section at 1423 K, a four-phase equilibrium region comprising Bcc(Cr), β-(Ni,Ru)Al, Al_8_Cr_5_, and Al_2_Ru, along with three three-phase regions, was identified. Complete mutual solubility between the NiAl and AlRu phases was achieved with approximately 10 at.% Cr. In the 55 at.% Ni section at 1173 K, two four-phase and seven three-phase equilibrium regions were observed. The addition of Cr was found to promote the emergence of the Fcc(Ni) + β-(Ni,Ru)Al + Ni_3_Al three-phase region and the Fcc(Ni) + β-(Ni,Ru)Al two-phase region. Critically, Cr addition enabled complete solubility between the β_1_ (NiAl) and β_2_ (AlRu) phases even at 1173 K. For the 60 at.% Ni section at 1423 K, while no four-phase equilibrium was found, two three-phase regions—(Ni,Ru)Al + Hcp(Ru) + Fcc(Ni) and (Ni,Ru)Al + Ni_3_Al + Fcc(Ni)—were confirmed. Notably, the (Ni,Ru)Al + Fcc(Ni) two-phase region exhibited a wide compositional range. This work provides essential experimental phase diagram data and insights for the design of Ni–Al–Cr–Ru-X high-entropy alloys and next-generation Ni-based superalloys.

## 1. Introduction

Ni-based superalloys are indispensable for high-temperature applications such as aerospace engine components and gas turbines, owing to their exceptional oxidation resistance and mechanical properties [1,2,3,4,5]. Aluminum is critical for forming the strengthening γ’ (Ni_3_Al) phase, while both Cr and Al are essential for high-temperature oxidation resistance. Recent studies have demonstrated that Ru additions can suppress the formation of deleterious topologically close-packed (TCP) phases, establishing Ru as a key element in fourth-generation single-crystal Ni-based superalloys [1,2,3,4,5]. Concurrently, aluminide coatings provide economical and effective surface protection for superalloys. Ru-modified aluminide coatings have been shown to inhibit the inward diffusion of Al and the outward diffusion of refractory elements, thereby suppressing the formation of the secondary reaction zone (SRZ) and preventing coating failure due to Al_2_O_3_ spallation [6,7]. Given the typical service temperatures of Ni-based superalloys (around 1050 °C, occasionally reaching 1150–1200 °C), a profound understanding of the phase relationships in the Ni–Al–Cr–Ru quaternary system is crucial for designing both advanced superalloys and Ru-modified aluminide coatings.

The Ni–Cr–Al ternary system has been extensively studied. Guan and Lin [8,9] experimentally determined several isothermal sections in the Ni-rich region. Investigations [10,11,12] focused on the Al-rich region, identifying three ternary compounds (τ_1_, τ_2_, τ_3_) and multiple invariant reactions. Thermodynamic assessments of this system have evolved, from the sub-regular solution models [13] to more sophisticated disordered–ordered models [14] and the cluster site approximation (CSA) model [15]. The most recent comprehensive assessment by Wang et al. [16] incorporated the composition range of the τ_1_ phase, providing calculated isothermal sections from 973 K to 1473 K.

The Ni–Cr–Ru ternary system presents relatively simpler phase relations. Early work by Chakravorty [17] proposed preliminary phase diagrams based on limited data. Subsequently, Zhu [18] established the first thermodynamic database for this system using CALPHAD extrapolation. More recently, Zhu et al. [19] employed a diffusion multiple method to obtain more accurate phase relations at 1073 K, 1173 K, 1273 K, and 1473 K, leading to an updated thermodynamic description.

The Ni–Al–Ru system is fundamental to understanding Ru roles in Ni–Al–Ru alloys [20]. Zhu et al. [21] combined experimental measurements and first-principles calculations to demonstrate that a two-phase equilibrium between NiAl and AlRu exists only in regions with Al content below 50 at.% at 1000 °C. Wang et al. [22,23] investigated Ru Local Atomic Structure and effect to γ’ evolution in Ni–Al–Ru alloys. A ternary phase, Al_14_Ni_2_Ru, was reported in the Al-rich corner [24,25], though its exact composition range remains debated. We previously investigated the Al–Ni–Ru ternary system at 1423 K and identified four three-phase regions and six primary solidification regions [26].

Experimental data for the Al–Cr–Ru ternary system are sparse. Compton et al. [27,28,29] determined partial isothermal sections at 600 °C and 1000 °C, identifying two ternary phases, τ_1_ and τ_2_.

Despite these efforts on constituent ternaries, experimental data for the quaternary Ni–Al–Cr–Ru system are extremely limited. Chakravorty [30] et al. investigated two isothermal sections at 1273 K and 1523 K for a constant 75 at.% Ni composition. However, this work was constrained by a lack of data for bounding ternary systems and relied on only four alloy compositions. Zhou et al. [31] investigated the temporal evolution of γ′ (L12) precipitates in a Ni–Al–Cr–Ru alloy using transmission electron microscopy and atom-probe tomography, revealing that Ru addition reduces the lattice misfit, accelerates compositional evolution, and influences elemental partitioning between γ and γ′ phases. Thermodynamic calculations by Zhu et al. [18] provided pseudo-ternary sections but lacked experimental validation. Therefore, to systematically investigate the phase equilibria of the Ni–Al–Cr–Ru system and elucidate the effect of Ru in both Ni-rich and Al–rich corners, three key constant-composition sections were selected based on practical relevance: the 55 at.% Al section at 1423 K (B2 stability in Al-rich region, relevant to Ru-modified aluminide coatings), the 55 at.% Ni section at 1173 K (Ni-rich corner at a typical superalloy service temperature), and the 60 at.% Ni section at 1423 K (high-temperature solution treatment range, where Fcc + B2 equilibria are of interest). This work systematically determines these pseudo-ternary isothermal sections at key temperatures and fixed compositions.

## 2. Materials and Methods

Twenty-six alloy samples (compositions listed in Table 1, Table 2 and Table 3) were prepared from high-purity elements (Ni, Cr, Al, Ru, 99.99 wt.%). Each 1 g sample was accurately weighed using an electronic balance and arc-melted under an argon atmosphere. To ensure homogeneity, each ingot was re-melted at least four times. The as-cast buttons were then sealed in evacuated quartz tubes and subjected to heat treatment at the target temperatures (1173 K or 1423 K). Annealing durations were selected based on prior studies [32]; alloys at 1173 K were annealed for 720 h and at 1423 K for 360 h. To confirm equilibrium, EDS measurements were taken from at least five regions per phase (standard deviations in Table 1, Table 2 and Table 3), and selected alloys (e.g., B3 and B6) annealed for 480 h and 720 h showed nearly identical microstructures (Supplementary Figure S1). Following annealing, the samples were quenched in ice water to preserve the high-temperature equilibrium microstructure.

Standard metallographic techniques were employed for sample preparation, involving grinding and polishing to obtain a smooth, scratch-free surface. Microstructural characterization was performed using an EVO MA10 scanning electron microscope (SEM, ZEISS, Oberkochen, Baden-Wurttemberg, Germany) equipped with an energy-dispersive spectrometer (EDS, Bruker Nano GmbH, Berlin, Germany) for phase morphology observation and compositional analysis. Phase identification was further confirmed by X-ray diffraction (XRD, Rigaku, Tokyo, Japan) using a Ultimate IV diffractometer with Cu Kα radiation.

## 3. Results

The phase equilibria of the Ni–Al–Cr–Ru quaternary system were experimentally determined for three key constant-composition sections at different temperatures. The nominal alloy compositions, the phases identified, and the corresponding phase compositions are summarized in Table 1, Table 2 and Table 3.

### 3.1. The 55 at.% Al Isothermal Section at 1423 K

**Figure 1:**
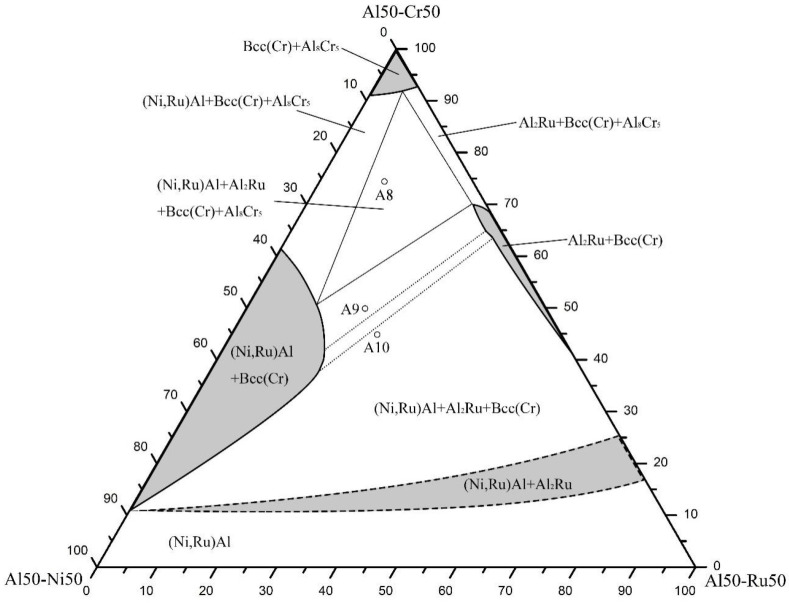
Experimentally determined 55 at.% Al isothermal section of the Ni–Al–Cr–Ru system at 1423 K, where the dashed lines are inferred from the phase relationships of the ternary boundaries.

To investigate phase equilibria involving the (Ni,Ru)Al phase with the B2 crystal structure, the 55 at.% Al constant-composition section within the 1423 K isothermal tetrahedron was selected. Ten alloys (A1-A10) were prepared. Their analyzed compositions, constituent phases, and phase compositions are listed in Table 1. The experimentally determined phase diagram is shown in Figure 1.

**Figure 2:**
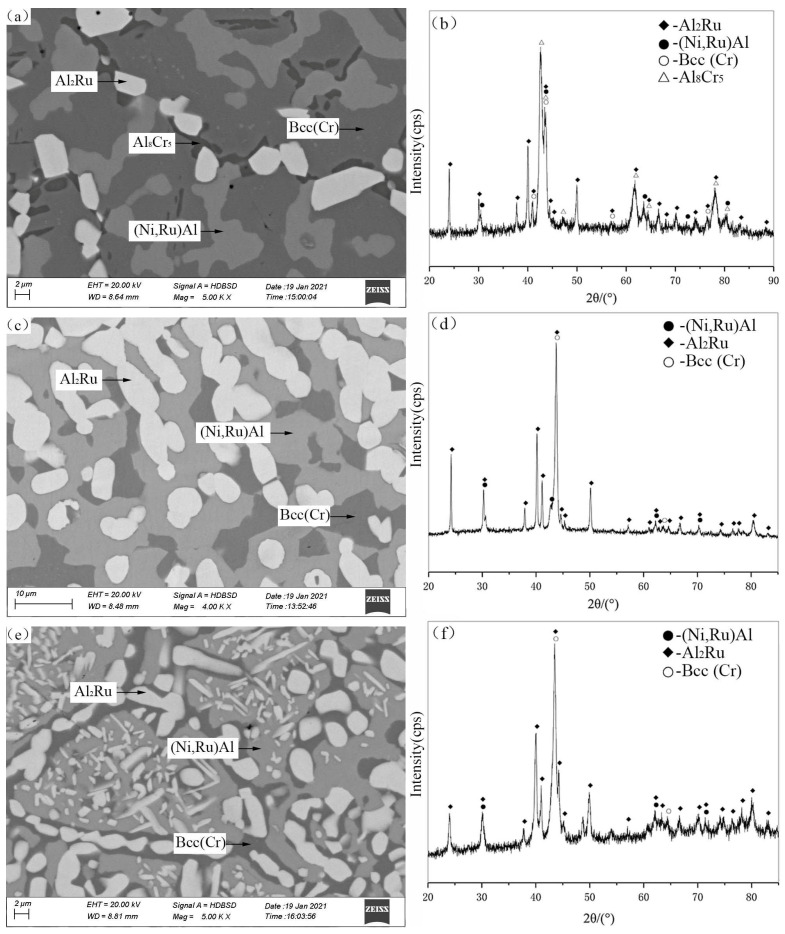
SEM images (**a**,**c**,**e**) and corresponding XRD patterns (**b**,**d**,**f**) for alloys A8 (**a**,**b**), A9 (**c**,**d**), and A10 (**e**,**f**) annealed at 1423 K. (**a**) A8: white Al_2_Ru, light gray (Ni,Ru)Al, black Al_8_Cr_5_, dark gray Bcc(Cr). (**c**) A9: white Al_2_Ru, light gray (Ni,Ru)Al, dark gray Bcc(Cr). (**e**) A10: white Al_2_Ru, light gray (Ni,Ru)Al, dark gray Bcc(Cr).

The microstructural analysis (Figure 2a, for alloy A8) and XRD patterns (Figure 2b) revealed a four-phase equilibrium region comprising Bcc(Cr), (Ni,Ru)Al, Al_8_Cr_5_, and Al_2_Ru. In this microstructure, the black Al_8_Cr_5_ phase, white blocky Al_2_Ru phase, and light-gray (Ni,Ru)Al phase are dispersed within the Bcc(Cr) matrix. The solid solubility of Cr and Ni in Al_2_Ru was measured to be up to 2.4 at.% and 0.3 at.%, respectively. The Al_8_Cr_5_ phase dissolved up to 1.4 at.% Ni and 3.3 at.% Ru. The Bcc(Cr) phase showed a remarkably high solubility for Al (up to 40.3 at.%), while the (Ni,Ru)Al phase dissolved up to 16.1 at.% Cr. Alloys A9 and A10 (Figure 2c–f) were located within the Al_2_Ru + Bcc(Cr) + (Ni,Ru)Al three-phase region, helping to define its compositional boundaries.

A key finding in this section is that with approximately 10 at.% Cr, the NiAl and AlRu phases become completely mutually soluble, forming a continuous β-(Ni,Ru)Al phase field.

### 3.2. The 55 at.% Ni Isothermal Section at 1173 K

**Figure 3:**
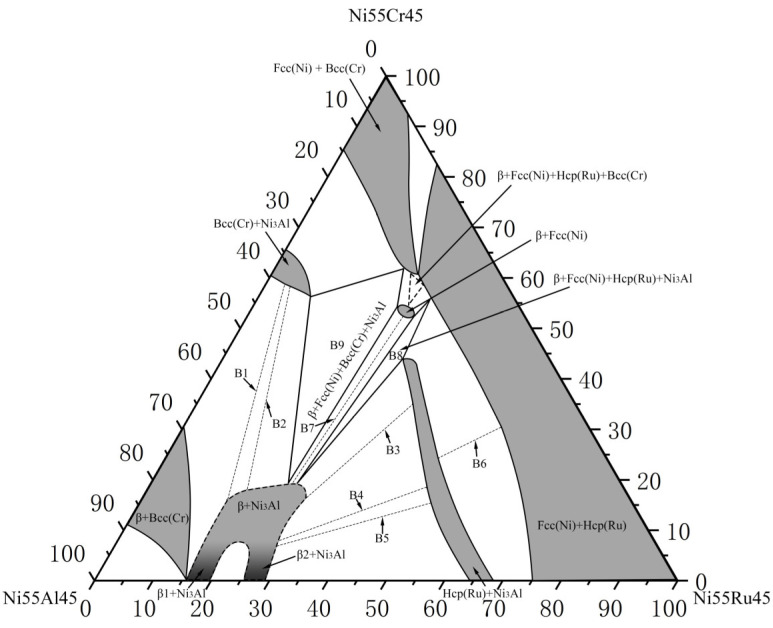
Experimentally determined 55 at.% Ni isothermal section of the Ni–Al–Cr–Ru system at 1173 K.

To examine the Ni-rich corner, nine alloys (B1–B9) were prepared for the 55 at.% Ni section at 1173 K. Table 2 lists their compositions and analyzed phase equilibria. The resulting phase diagram is shown in Figure 3.

**Figure 4:**
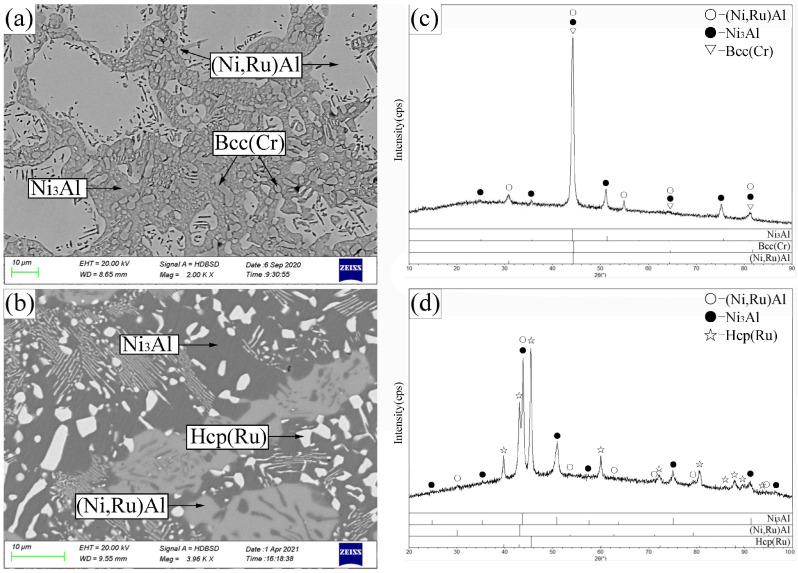
SEM images (**a**,**b**) and corresponding XRD patterns (**c**,**d**) for alloys B1 (**a**,**c**) and B3 (**b**,**d**) annealed at 1173 K. (**a**) B1: large gray β-(Ni,Ru)Al, recessed gray Ni_3_Al, island-shaped dark-gray Bcc(Cr). (**b**) B3: bright Hcp(Ru), light-gray β-(Ni,Ru)Al, dark-gray Ni_3_Al.

Alloy B1 (Figure 4a,c) exhibited a three-phase equilibrium of Bcc(Cr) + β-(Ni,Ru)Al + Ni_3_Al. The large gray blocks are β-(Ni,Ru)Al, surrounded by the Ni_3_Al phase (recessed due to etching), with the Bcc(Cr) phase appearing as protruding, dotted features. The Ni_3_Al phase dissolved up to 6.9 at.% Cr and 0.3 at.% Ru, while the Bcc(Cr) phase contained 4.5 at.% Ni, 5.1 at.% Ru, and negligible Al.

**Figure 5:**
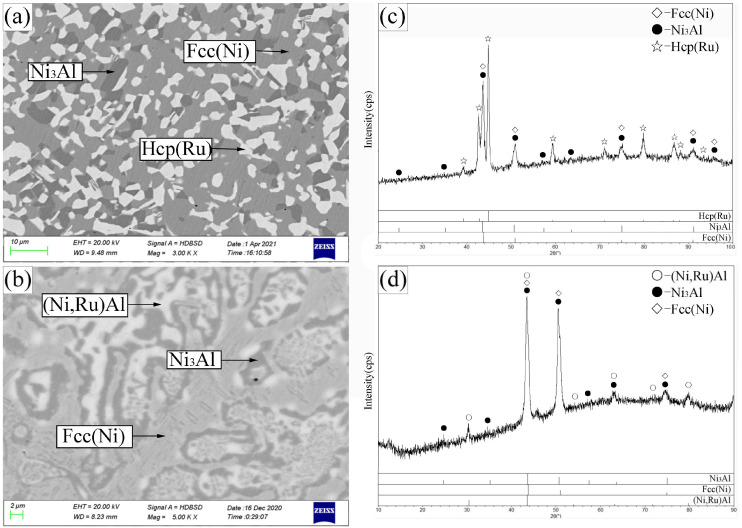
SEM images (**a**,**b**) and corresponding XRD patterns (**c**,**d**) for alloys B6 (**a**,**c**) and B7 (**b**,**d**) annealed at 1173 K. (**a**) B6: bright Hcp(Ru), light-gray Fcc(Ni), dark-gray Ni_3_Al. (**b**) B7: light-gray Fcc(Ni), dark-gray Ni_3_Al, bright β-(Ni,Ru)Al.

Alloys B3–B5 (B3 shown in Figure 4b,d) were located in the Hcp(Ru) + β-(Ni,Ru)Al + Ni_3_Al three-phase region. The Hcp(Ru) phase showed extensive solubility for both Ni (up to 37.7 at.%) and Cr (up to 32.1 at.%). Alloy B6 (Figure 5a,c) defined the Hcp(Ru) + Fcc(Ni) + Ni_3_Al three-phase region, while alloy B7 (Figure 5b,d) established the Ni_3_Al + Fcc(Ni) + β-(Ni,Ru)Al three-phase equilibrium.

**Figure 6:**
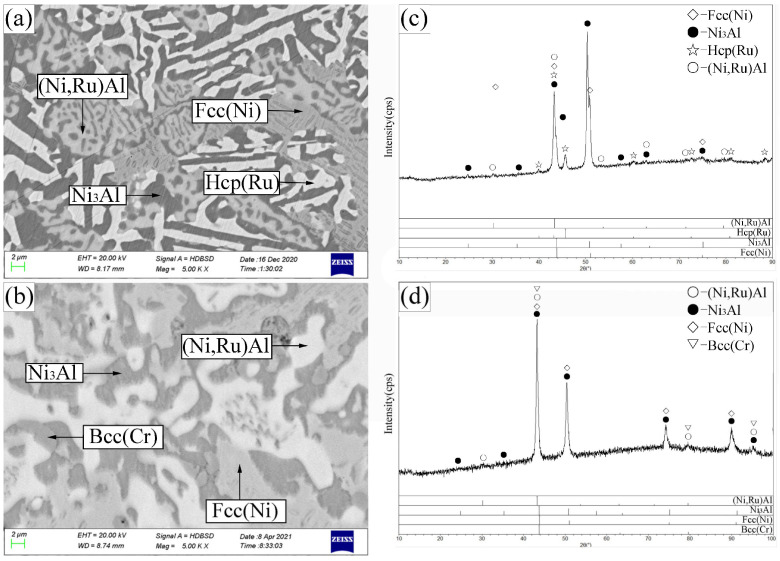
SEM images (**a**,**b**) and corresponding XRD patterns (**c**,**d**) for alloys B8 (**a**,**c**) and B9 (**b**,**d**) annealed at 1173 K. (**a**) B8: bright Hcp(Ru), light-gray β-(Ni,Ru)Al, gray Fcc(Ni), dark-gray Ni_3_Al. (**b**) B9: bright β-(Ni,Ru)Al, light-gray Fcc(Ni), recessed gray Ni_3_Al, raised gray Bcc(Cr).

Crucially, alloys B8 and B9 revealed two distinct four-phase equilibria. Alloy B8 (Figure 6a,c) was located in the Fcc(Ni) + β-(Ni,Ru)Al + Hcp(Ru) + Ni_3_Al region. Alloy B9 (Figure 6b,d) defined the Fcc(Ni) + β-(Ni,Ru)Al + Bcc(Cr) + Ni_3_Al four-phase equilibrium. The identification of the Fcc(Ni) + β-(Ni,Ru)Al two-phase region and the associated Fcc(Ni) + β-(Ni,Ru)Al + Ni_3_Al three-phase region, which are absent in the bounding ternary systems, demonstrates the significant influence of Cr. Furthermore, the absence of a two-phase region between β_1_ (NiAl) and β_2_ (AlRu) in any alloy suggests that the addition of Cr promotes complete mutual solubility between these phases even at 1173 K, similar to its effect in the Al–Cr–Ru system.

Based on these results, the 55 at.% Ni isothermal section at 1173 K (Figure 3) contains two experimentally confirmed four-phase regions (Fcc(Ni) + β-(Ni,Ru)Al + Hcp(Ru) + Ni_3_Al and Fcc(Ni) + β-(Ni,Ru)Al + Bcc(Cr) + Ni_3_Al) Fcc(Ni)Fcc(Ni)Bcc(Cr)Fcc(Ni)Bcc(Cr) and seven three-phase regions.

### 3.3. The 60 at.% Ni Isothermal Section at 1423 K

**Figure 7:**
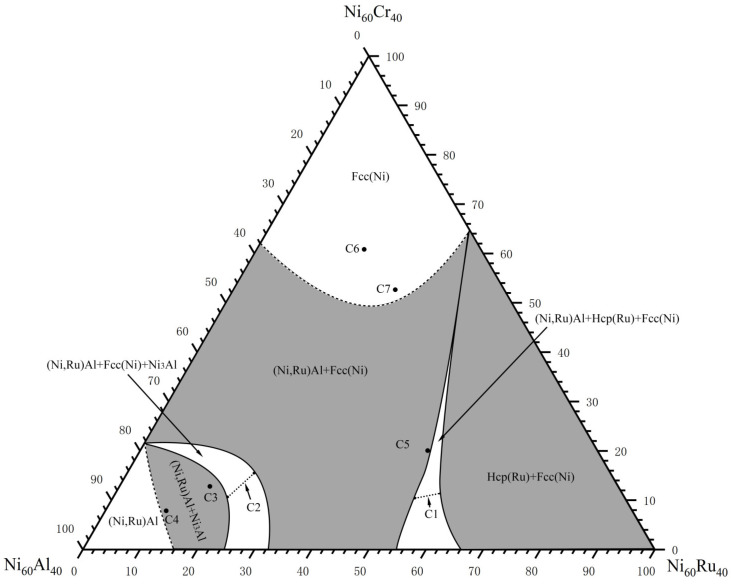
Experimentally determined 60 at.% Ni isothermal section of the Ni–Al–Cr–Ru. system at 1423 K.

Seven alloys (C1–C7) were prepared to determine the 60 at.% Ni section at 1423 K. Table 3 lists their compositions and phase equilibria, with the resulting phase diagram shown in Figure 7.

**Figure 8:**
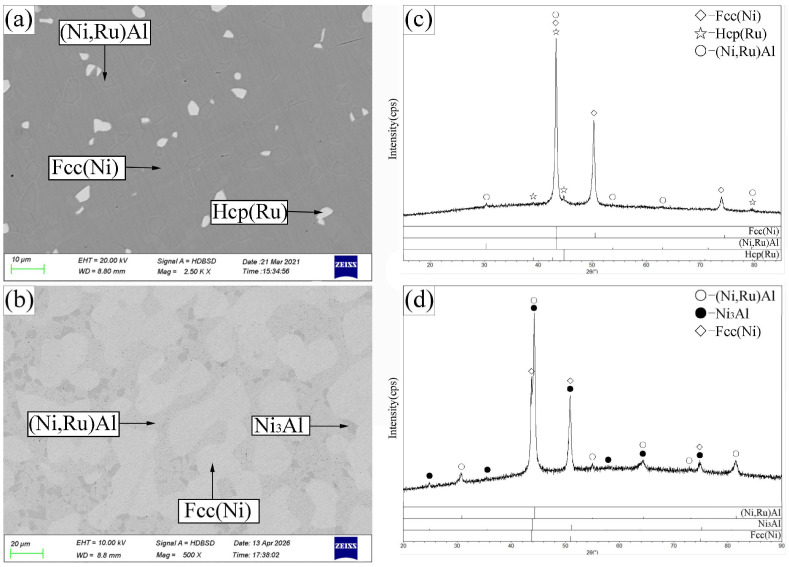
SEM images (**a**,**b**) and corresponding XRD patterns (**c**,**d**) for alloys C1 (**a**,**c**) and C2 (**b**,**d**) annealed at 1423 K. (**a**) C1: bright Hcp(Ru), recessed gray (Ni,Ru)Al, raised gray Fcc(Ni). (**b**) C2: light-gray (Ni,Ru)Al, gray Fcc(Ni), dark-gray Ni_3_Al.

**Figure 9:**
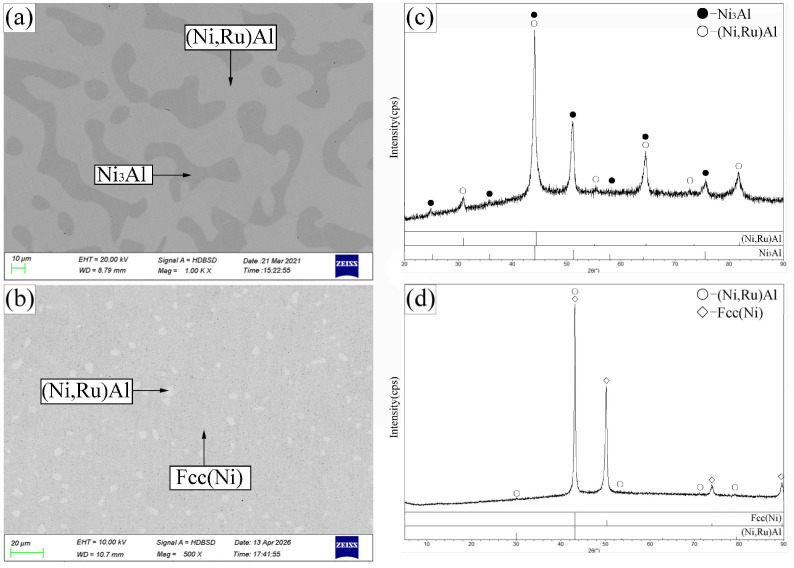
SEM images (**a**,**b**) and corresponding XRD patterns (**c**,**d**) for alloys C3 (**a**,**c**) and C5 (**b**,**d**) annealed at 1423 K. (**a**) C3: light-gray (Ni,Ru)Al, dark-gray Ni_3_Al. (**b**) C5: light-gray (Ni,Ru)Al, dark-gray Fcc(Ni).

**Figure 10:**
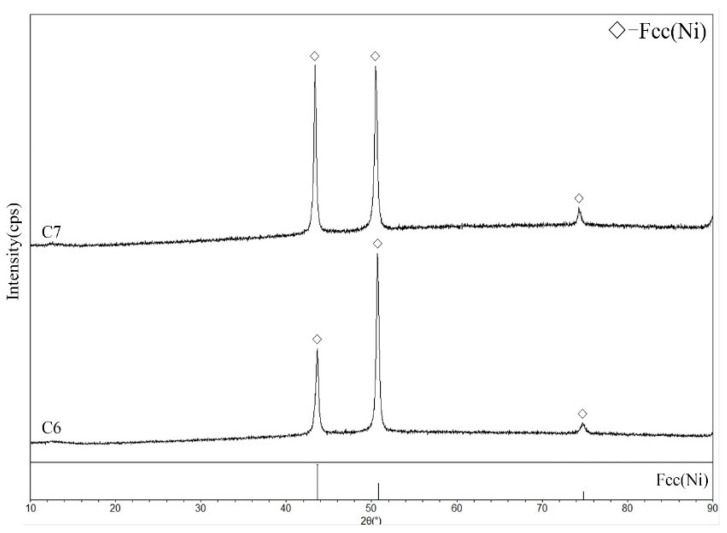
XRD patterns for single-phase Fcc(Ni) alloys C6 and C7, annealed at 1423 K.

Alloy C1 (Figure 8a,c) was located in the (Ni,Ru)Al + Hcp(Ru) + Fcc(Ni) three-phase region. Alloy C2 (Figure 8b,d) defined the (Ni,Ru)Al + Ni_3_Al + Fcc(Ni) three-phase region. Alloys C3 and C4 were found to be within the (Ni,Ru)Al + Ni_3_Al two-phase region, while alloy C5 (Figure 9b,d) resided in the (Ni,Ru)Al + Fcc(Ni) two-phase region. Notably, the Fcc(Ni) phase in alloy C5 contains 19.9 at.% Ru, a relatively high value. This is consistent with the Ni-Ru binary phase diagram [32], which indicates that the Fcc(Ni) phase can dissolve over 20 at.% Ru at 1423 K. Similarly, alloy C1 (21.7 at.% Ru nominal) also exhibits a high Ru content in its Fcc(Ni) phase (20.6 at.%). In contrast, alloys with lower Ru contents-such as C2 (8.9 at.% Ru), C6 (8.4 at.% Ru), and C7 (9.4 at.% Ru)-show correspondingly lower Ru concentrations in their Fcc(Ni) phases (5.4, 8.4, and 9.4 at.%, respectively). This trend confirms that the Ru content in Fcc(Ni) is governed by the overall alloy composition and established phase equilibria. Alloys C6 and C7 (Figure 10) were single-phase Fcc(Ni).

No four-phase equilibrium was found in this section. The two experimentally confirmed three-phase regions are (Ni,Ru)Al + Hcp(Ru) + Fcc(Ni) and (Ni,Ru)Al + Ni_3_Al + Fcc(Ni). The (Ni,Ru)Al + Fcc(Ni) two-phase region exhibits a particularly wide compositional range. Due to the limited number of alloys, the boundaries of some phase regions (e.g., the single-phase Fcc(Ni) and (Ni,Ru)Al fields) are indicated with dashed lines in Figure 7, representing our best estimate.

## 4. Discussion

This study provides the first systematic experimental investigation of three critical isothermal sections within the Ni–Al–Cr–Ru quaternary system. The results offer significant insights into the role of Cr and Ru in modifying phase equilibria, which are directly relevant to the design of advanced Ni-based superalloys and high-entropy alloys.

In the 55 at.% Al section at 1423 K, the complete mutual solubility between NiAl and AlRu upon the addition of ~10 at.% Cr is a key finding. This aligns with the observations in the ternary Al–Cr–Ru system [27,28,29] where Cr expanded the β-(AlRu) phase field, and suggests that Cr acts as a strong stabilizer for the continuous B2 phase field. The existence of the Bcc(Cr) + β-(Ni,Ru)Al + Al_8_Cr_5_ + Al_2_Ru four-phase equilibrium delineates the complex interactions between the B2, Bcc, and Al-rich intermetallic phases in this Al-rich corner.

The 55 at.% Ni section at 1173 K is particularly illuminating for understanding phase stability in the Ni-rich region relevant to superalloy matrices. The most striking observation is the emergence of the Fcc(Ni) + β-(Ni,Ru)Al two-phase region and the associated Fcc(Ni) + β-(Ni,Ru)Al + Ni_3_Al three-phase region, which are absent in the Ni–Cr–Al, Ni–Al–Ru, and Ni–Cr–Ru ternary boundaries at this temperature and Ni content. This unequivocally demonstrates that Cr addition fundamentally alters the phase equilibria, stabilizing the coexistence of the Fcc matrix with the B2 phase and the γ’ (Ni_3_Al) phase.

In addition to the two experimentally confirmed four-phase regions, the phase relationships in this section suggest the existence of a third four-phase region: Fcc(Ni) + Bcc(Cr) + Hcp(Ru) + β-(Ni,Ru)Al. This inference is based on the experimentally determined equilibria of alloys B8 and B9, combined with the known phase relationships in the three bounding ternary systems (Ni–Al–Ru, Ni–Cr–Ru, and Ni–Cr–Al) at 1173 K, as well as the phase rule. While this region is proposed as a logical deduction from the available data, its experimental confirmation remains a task for future work.

This finding is particularly significant in the context of Ni-based superalloys. While Alloy 718, the most widely used Ni-based superalloy, relies primarily on γ″ (Ni_3_Nb) precipitation for strengthening, many other advanced Ni-based superalloys (e.g., René 88, CMSX-4) utilize γ′ (Ni_3_Al) as the primary strengthening phase. Our results provide fundamental phase equilibria data that are essential for designing γ′-strengthened alloys with improved microstructural stability, particularly when Ru and Cr are present. Moreover, recent studies on high-entropy superalloys have demonstrated that the synergy between ordered L12 and disordered Fcc phases can achieve exceptional strength–ductility combinations [33]. Our phase stability results, showing the coexistence of γ′ (L12) with Fcc(Ni) and β-(Ni,Ru)Al, offer a valuable basis for tailoring such heterostructures in compositionally complex alloys. Furthermore, the absence of any observed miscibility gap between β_1_ (NiAl) and β_2_ (AlRu) in this section strongly supports the hypothesis that Cr promotes complete intersolubility, effectively merging what were separate B2 phases in the ternary Ni–Al–Ru system into a single, chemically complex β-(Ni,Ru,Cr)Al phase field at 1173 K. This reconciles the previously contradictory reports on NiAl/AlRu miscibility [21,22,23] by highlighting the critical influence of a third element like Cr.

Beyond the context of superalloys, our phase stability results also offer insights into the design of B2-strengthened high-entropy alloys. FeMnAlCNi low-density steels, for instance, achieve strengthening through B2 intermetallic precipitates [34]. The present results demonstrate that Cr promotes the stability of a continuous B2 phase field in the Ni–Al–Cr–Ru system, and the established isothermal sections reveal the compositional windows where B2 coexists with Fcc or L12 phases. This provides a pathway for designing two-phase (L12 + B2 or Fcc + B2) microstructures in high-entropy superalloys. Controlled heat treatments based on these experimentally determined phase equilibria could enable the precipitation of coherent B2 nanoparticles within Fcc/L12 matrices, potentially leading to enhanced strength–ductility synergy.

The 60 at.% Ni section at 1423 K, a temperature approaching the solution treatment range for many superalloys, reveals relatively simpler equilibria. The absence of four-phase regions suggests a gradual transition of phase fields. The wide (Ni,Ru)Al + Fcc(Ni) two-phase region is a highly desirable feature for alloy design, as it indicates a broad composition window where a high volume fraction of a strengthening B2 phase can coexist with the ductile Fcc matrix. The single-phase Fcc(Ni) field at high Cr + Ru contents (alloys C6, C7) also provides valuable information for solid-solution strengthening.

The results presented herein also hold significant promise for combinatorial alloy design, particularly in the context of additively manufactured high-entropy superalloys. The isothermal sections established in this work can serve as a high-throughput screening tool for selecting alloy compositions with targeted phase constitutions (e.g., γ/γ′ two-phase region, Fcc + B2 two-phase region) prior to additive manufacturing feedstock development. Furthermore, the phase stability data as a function of Cr and Ru additions can guide the design of composition gradients in combinatorial additive manufacturing experiments, enabling rapid exploration of composition-microstructure- property relationships. This approach could significantly accelerate the discovery of novel superalloys with optimized phase balances for high-temperature performance.

Overall, these experimental results provide a robust foundation for refining CALPHAD thermodynamic databases of the Ni–Al–Cr–Ru system. The new data on phase compositions and multi-phase equilibria will enable more accurate predictions of phase stability in complex, multi-component alloys. Future work should focus on extending this experimental investigation to other constant-composition sections and temperatures to build a complete quaternary phase diagram. Thermodynamic modeling incorporating these new data is currently underway and will be reported separately.

## 5. Conclusions

The phase equilibria of the Ni–Al–Cr–Ru quaternary system were experimentally investigated using the equilibrated alloy method for three key isothermal sections. The main findings are:

For 55 at.% Al at 1423 K: One four-phase equilibrium region, Bcc(Cr) + β-(Ni,Ru)Al + Al_8_Cr_5_ + Al_2_Ru, and three three-phase regions were identified. Complete mutual solubility between the NiAl and AlRu phases is achieved with approximately 10 at.% Cr in this section.

For 55 at.% Ni at 1173 K: Three four-phase equilibrium regions (Fcc(Ni) + β-(Ni,Ru)Al + Hcp(Ru) + Ni_3_Al, Fcc(Ni) + β-(Ni,Ru)Al + Bcc(Cr) + Ni_3_Al, and Fcc(Ni) + Bcc(Cr) + Hcp(Ru) + β-(Ni,Ru)Al) and seven three-phase regions were identified. Cr addition promotes the formation of the Fcc(Ni) + β-(Ni,Ru)Al + Ni_3_Al three-phase region and the Fcc(Ni) + β-(Ni,Ru)Al two-phase region. Crucially, Cr enables complete mutual solubility between the β_1_ (NiAl) and β_2_ (AlRu) phases even at 1173 K.

For 60 at.% Ni at 1423 K: No four-phase equilibria were found. Two three-phase regions, (Ni,Ru)Al + Hcp(Ru) + Fcc(Ni) and (Ni,Ru)Al + Ni_3_Al + Fcc(Ni), were identified. The (Ni,Ru)Al + Fcc(Ni) two-phase region exhibits a notably wide compositional range.

This work provides essential experimental phase diagram data that are critical for the design of Ni–Al–Cr–Ru-based high-entropy alloys and next-generation Ni-based superalloys, and serves as a key benchmark for future thermodynamic modeling.

## Figures and Tables

**Table 1:** Nominal and phase compositions of alloys in the 55 at.% Al isothermal section of the Ni–Al–Cr–Ru system at 1423 K.

Alloy No.	Alloy Composition (at. %)				Phase	Phase Composition (at. %)			
	Ni	Al	Cr	Ru		Ni	Al	Cr	Ru
A1	22.0	55.7	10.1	12.2	Al_2_Ru	3.7 ± 0.54	64.7 ± 0.28	1.8 ± 0.23	29.8 ± 0.49
					(Ni,Ru)Al	26.1 ± 0.13	53.4 ± 0.09	11.6 ± 0.09	8.9 ± 0.14
A2	7.7	50.6	34.7	7.0	Al_2_Ru	0.4 ± 0.05	65.4 ± 0.13	1.8 ± 0.28	32.4 ± 0.2
					(Ni,Ru)Al	19.6 ± 0.02	53.9 ± 0.3	19.5 ± 0.27	7.0 ± 0.04
					Bcc (Cr)	3.3 ± 0.04	44.9 ± 0.17	49.6 ± 0.16	2.2 ± 0.03
A3	14.8	53.2	25.4	6.6	Al_2_Ru	0.8 ± 0.13	65.7 ± 0.21	1.1 ± 0.14	32.4 ± 0.15
					(Ni,Ru)Al	19.6 ± 0.14	54.0 ± 0.25	19.4 ± 0.13	7.0 ± 0.05
					Bcc(Cr)	2.9 ± 0.35	44.9 ± 0.23	50.1 ± 0.25	2.1 ± 0.04
					Al_8_Cr_5_	2.9 ± 0.14	58.1 ± 0.18	35.5 ± 0.2	3.5 ± 0.04
A4	8.7	55.8	21.5	14.0	Al_2_Ru	0.4 ± 0.07	66.2 ± 0.18	0.9 ± 0.19	32.5 ± 0.24
					(Ni,Ru)Al	19.8 ± 0.39	54.2 ± 0.28	18.9 ± 0.34	7.1 ± 0.16
					Bcc(Cr)	3.4 ± 0.13	45.2 ± 0.29	49.1 ± 0.21	2.3 ± 0.04
A5	2.3	54.5	40.9	2.3	Bcc(Cr)	2.3 ± 0.1	43.1 ± 0.3	53.3 ± 0.21	1.3 ± 0.01
					Al_8_Cr_5_	2.3 ± 0.11	57.7 ± 0.09	37.5 ± 0.2	2.5 ± 0.01
A6	15.0	50.3	26.6	8.1	Al_2_Ru	0.9 ± 0.13	65.0 ± 0.13	1.3 ± 0.14	32.8 ± 0.14
					(Ni,Ru)Al	23.8 ± 0.7	53.7 ± 0.36	14.4 ± 0.85	8.1 ± 0.22
					Bcc(Cr)	2.0 ± 0.44	39.9 ± 0.06	56.6 ± 0.31	1.5 ± 0.07
A7	15.1	50.0	26.9	8.0	Al_2_Ru	1.2 ± 0.04	65.1 ± 0.09	1.0 ± 0.3	32.7 ± 0.24
					(Ni,Ru)Al	23.0 ± 0.56	53.7 ± 0.04	15.1 ± 0.49	8.2 ± 0.02
					Bcc(Cr)	1.7 ± 0.03	40.1 ± 0.13	56.9 ± 0.2	1.3 ± 0.1
A8	6.0	46.5	41.5	6.0	Al_2_Ru	0.3 ± 0.12	64.7 ± 0.34	2.4 ± 0.25	32.6 ± 0.03
					(Ni,Ru)Al	24.0 ± 0.0	52.8 ± 0.02	16.1 ± 0.02	7.1 ± 0.01
					Bcc(Cr)	1.7 ± 0.0	40.3 ± 0.22	56.5 ± 0.34	1.5 ± 0.13
					Al_8_Cr_5_	1.4 ± 0.14	56.5 ± 0.81	38.8 ± 0.79	3.3 ± 0.62
A9	11.9	53.1	19.9	15.1	Al_2_Ru	1.4 ± 0.25	64.6 ± 0.5	2.1 ± 0.92	31.9 ± 1.18
					(Ni,Ru)Al	24.3 ± 0.21	52.8 ± 0.44	12.9 ± 0.98	10.0 ± 0.34
					Bcc(Cr)	1.4 ± 0.3	36.2 ± 0.17	60.9 ± 0.0	1.5 ± 0.04
A10	10.3	51.8	22.6	15.4	Al_2_Ru	0.5 ± 0.1	64.1 ± 0.07	3.5 ± 0.05	31.9 ± 0.11
					(Ni,Ru)Al	25.2 ± 0.02	52.5 ± 0.04	11.8 ± 0.03	10.5 ± 0.04
					Bcc(Cr)	1.9 ± 0.11	35.0 ± 0.12	61.7 ± 0.13	1.4 ± 0.12

**Table 2:** Nominal and phase compositions of alloys in the 55 at.% Ni isothermal section of the Ni–Al–Cr–Ru system at 1173 K.

Alloy No.	Alloy Composition (at. %)	Phase	Phase Composition (at. %)			
			Ni	Al	Cr	Ru
B1	Ni49.6Al30.8Cr12.1Ru7.5	Ni_3_Al	70.5 ± 0.32	22.3 ± 0.50	6.9 ± 0.26	0.3 ± 0.09
		β(Ni,Ru)Al	46.2 ± 0.03	36.3 ± 0.6	7.3 ± 0.84	10.2 ± 0.46
		Bcc(Cr)	4.5 ± 0.45	0.5 ± 0.22	89.9 ± 0.56	5.1 ± 0.1
B2	Ni48.7Al29.4Cr13.0Ru8.9	Ni_3_Al	69.2 ± 0.08	21.6 ± 0.06	8.5 ± 0.09	0.7 ± 0.07
		β(Ni,Ru)Al	39.9 ± 0.27	37.1 ± 0.07	7.9 ± 0.09	15.1 ± 0.25
		Bcc(Cr)	6.6 ± 0.14	0.2 ± 0.34	86.9 ± 0.61	6.3 ± 0.15
B3	Ni55Al18Cr13.5Ru13.5	Ni_3_Al	70.7 ± 0.15	18.9 ± 0.03	7.5 ± 0.17	2.9 ± 0.36
		β(Ni,Ru)Al	18.2 ± 0.56	36.7 ± 0.09	11.4 ± 0.37	33.7 ± 0.19
		Hcp(Ru)	37.7 ± 0.08	2.7 ± 0.58	32.1 ± 0.45	27.6 ± 0.38
B4	Ni54.4Al12.5Cr15.5Ru17.6	Ni_3_Al	70.4 ± 0.06	20.0 ± 0.24	6.0 ± 0.13	3.6 ± 0.42
		β(Ni,Ru)Al	19.0 ± 0.34	36.7 ± 0.56	10.6 ± 0.11	33.7 ± 0.59
		Hcp(Ru)	32.5 ± 0.45	1.8 ± 0.43	30.0 ± 0.93	35.7 ± 0.96
B5	Ni48.7Al27.1Cr5.5Ru18.7	Ni_3_Al	72.6 ± 0.11	22.2 ± 0.19	2.9 ± 0.12	2.3 ± 0.18
		β(Ni,Ru)Al	19.3 ± 0.12	42.7 ± 0.07	4.8 ± 0.03	33.2 ± 0.03
		Hcp(Ru)	19.7 ± 0.68	1.4 ± 0.15	19.1 ± 0.11	59.8 ± 0.91
B6	Ni55.6Al10.8Cr11.2Ru22.4	Ni_3_Al	70.1 ± 1.04	19.2 ± 0.10	5.7 ± 0.32	5.0 ± 0.82
		Hcp(Ru)	25.7 ± 0.53	0.9 ± 0.33	21.2 ± 0.36	52.2 ± 0.94
		Fcc(Ni)	69.7 ± 0.12	9.8 ± 0.12	9.9 ± 0.06	10.6 ± 0.05
B7	Ni53.9Al19.4Cr18.1Ru8.6	Ni_3_Al	69.8 ± 0.53	20.3 ± 0.11	8.6 ± 0.22	1.3 ± 0.34
		Fcc(Ni)	56.3 ± 0.45	6.6 ± 0.11	30.9 ± 0.13	6.2 ± 0.21
		β(Ni,Ru)Al	26.3 ± 0.44	39.7 ± 0.21	8.9 ± 0.05	25.1 ± 0.31
B8	Ni55.3Al15.8Cr15.8Ru13.1	Ni_3_Al	70.2 ± 0.29	19.7 ± 0.08	7.6 ± 0.19	2.5 ± 0.19
		Hcp(Ru)	39.3 ± 0.09	2.7 ± 0.25	32.5 ± 0.14	25.5 ± 0.23
		β(Ni,Ru)Al	21.6 ± 0.24	36.6 ± 0.17	11.2 ± 0.01	30.5 ± 0.06
		Fcc(Ni)	56.9 ± 0.39	7.0 ± 0.09	24.2 ± 0.07	11.9 ± 0.41
B9	Ni55Al13Cr9.5Ru22.5	Ni_3_Al	69.4 ± 0.15	20.2 ± 0.06	8.2 ± 0.74	2.2 ± 0.39
		Bcc(Cr)	6.2 ± 0.34	0.5 ± 0.11	83.2 ± 0.16	10.1 ± 0.07
		β(Ni,Ru)Al	22.3 ± 0.48	38.2 ± 0.23	9.8 ± 0.22	29.7 ± 0.17
		Fcc(Ni)	57.1 ± 0.68	7.6 ± 0.59	25.4 ± 0.47	9.9 ± 0.81

**Table 3:** Nominal and phase compositions of alloys in the 60 at.% Ni isothermal section of the Ni–Al–Cr–Ru system at 1423 K.

Alloy No.	Alloy Composition (at. %)	Phase	Phase Composition (at. %)			
			Ni	Al	Cr	Ru
C1	Ni58.4Al15.5Cr4.1Ru21.7	Hcp(Ru)	21.5 ± 0.15	3.2 ± 0.21	10.1 ± 0.12	65.2 ± 0.23
		(Ni,Ru)Al	39.0 ± 0.82	31.2 ± 0.32	3.1 ± 0.24	26.7 ± 1.27
		Fcc(Ni)	61.9 ± 0.45	13.2 ± 0.19	4.3 ± 0.05	20.6 ± 0.29
C2	Ni60.0Al25.4Cr5.7Ru8.9	Ni_3_Al	71.8 ± 0.17	21.0 ± 0.22	4.5 ± 0.16	2.7 ± 0.34
		(Ni,Ru)Al	51.4 ± 0.13	32.6 ± 0.19	4.1 ± 0.07	11.9 ± 0.19
		Fcc(Ni)	70.0 ± 0.90	16.0 ± 0.57	8.6 ± 0.46	5.4 ± 0.18
C3	Ni60.8Al28.2Cr4.8Ru6.2	Ni_3_Al	71.3 ± 0.04	21.5 ± 0.08	5.2 ± 0.08	2.0 ± 0.04
		(Ni,Ru)Al	55.8 ± 0.28	31.1 ± 0.11	5.1 ± 0.15	8.0 ± 0.24
C4	Ni58.4Al33.2Cr3.0Ru5.4	Ni_3_Al	71.9 ± 0.10	24.3 ± 0.04	3.0 ± 0.04	0.8 ± 0.03
		(Ni,Ru)Al	59.3 ± 0.22	33.0 ± 0.05	3.2 ± 0.03	4.5 ± 0.19
C5	Ni58.7Al13.3Cr8.0Ru20.0	Fcc(Ni)	60.6 ± 0.09	11.4 ± 0.09	8.1 ± 0.12	19.9 ± 0.13
		(Ni,Ru)Al	19.1 ± 0.04	39.7 ± 0.19	4.3 ± 0.13	36.9 ± 0.10
C6	Ni59.2Al8.1Cr24.3Ru8.4	Fcc(Ni)	59.2	8.1	24.3	8.4
C7	Ni61.5Al8.0Cr21.1Ru9.4	Fcc(Ni)	61.5	8.0	21.1	9.4

## Data Availability

The original contributions presented in this study are included in the article/Supplementary Material. Further inquiries can be directed to the corresponding authors.

## Supplementary Materials

The following supporting information can be downloaded at: https://www.mdpi.com/article/10.3390/ma19081669/s1. Figure S1: SEM images for alloys B3 (a,b) and B6 (c,d) annealed at 1173 K: (a,c): Annealed for 480 h; (b,d): Annealed for 720 h.

## Abbreviations

The following abbreviations are used in this manuscript:

Bcc	Body-Centered Cubic
CALPHAD	Calculation of Phase Diagrams
EDS	Energy-Dispersive Spectroscopy
Fcc	Face-Centered Cubic
Hcp	Hexagonal Close-Packed
SEM	Scanning Electron Microscopy
SRZ	Secondary Reaction Zone
TCP	Topologically Close-Packed
XRD	X-ray Diffraction
CSA	Cluster Site Approximation
