# Supplementary figures and images for: Experimental Determination of Isothermal Sections in the Ni–Al–Cr–Ru Quaternary System: Implications for Ni-Based Superalloys and High-Entropy Alloys
Source: Materials (Basel). 2026 Apr 21;19(8):1669. doi: 10.3390/ma19081669 (PMC13118003; doi:10.3390/ma19081669)

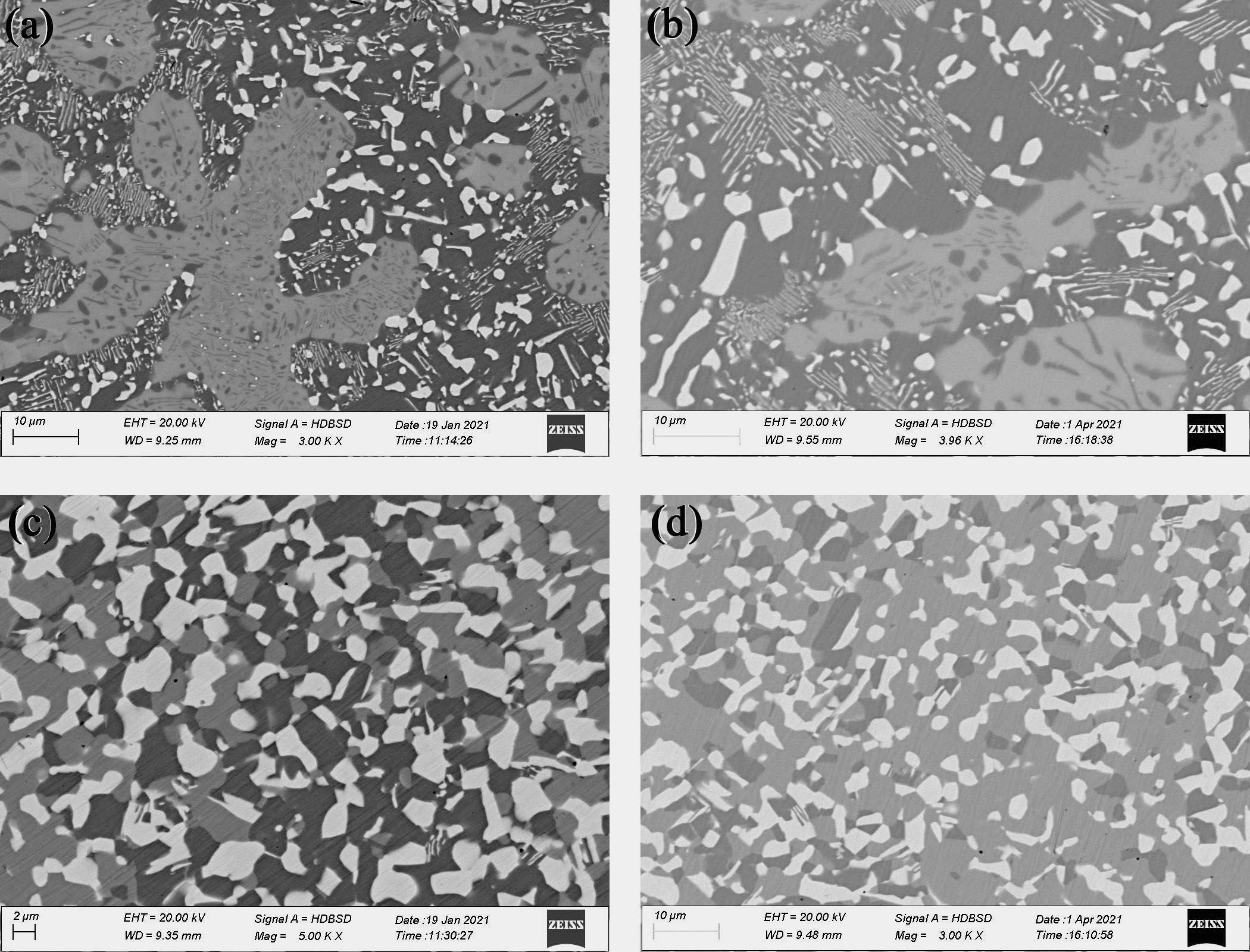

Supplement: Supplementary file 1 [file materials-19-01669-s001.zip › materials-4205110-Figure S1.jpg]
